# Multi‐omics analysis of tumor mutational burden combined with prognostic assessment in epithelial ovarian cancer based on TCGA database

**DOI:** 10.7150/ijms.50491

**Published:** 2020-10-23

**Authors:** Jinhui Liu, Wei Xu, Siyue Li, Rui Sun, Wenjun Cheng

**Affiliations:** Department of Gynecology, the First Affiliated Hospital of Nanjing Medical University, Nanjing, Jiangsu Province, People's Republic of China.

**Keywords:** epithelial ovarian cancer, tumor mutational burden, prognostic signature, immune infiltrates

## Abstract

**Background:** Tumor mutation burden (TMB) is considered as a novel biomarker of response to immunotherapy and correlated with survival outcomes in various malignancies. Here, TMB-related genes (TRGs) expression signatures were constructed to investigate the association between TMB and prognosis in epithelial ovarian cancer (EOC), and the potential mechanism in immunoregulation was also explored.

**Methods:** Based on somatic mutation data of 436 EOC samples from The Cancer Genome Atlas database, we examined the relationship between TMB level and overall survival (OS), as well as disease-free survival (DFS). Next, the TRGs signatures were constructed and validated. Differential abundance of immune cell infiltration, expression levels of immunomodulators and functional enrichment in high- and low-risk groups were also analyzed.

**Results:** Higher TMB level revealed better OS and DFS, and correlated with earlier clinical stages in EOCs (*P* = 2.796e-04). The OS-related prognostic model constructed based on seven TRGs (*B3GALT1, LIN7B, ANGPT2, D2HGDH, TAF13, PFDN4* and *DNAJC19*) significantly stratified EOC patients into high- and low-risk groups (*P <* 0.001). The AUC values of the seven-gene prognostic signature at 1 year, 3 years, and 5 years were 0.703, 0.758 and 0.777. While the DFS-related prognostic model was constructed based on the 4 TRGs (*LPIN3, PXYLP1, IGSF23* and *B3GALT1*), with AUCs of 0.617, 0.756, and 0.731, respectively. Functional analysis indicated that immune‐related pathways were enriched in low‐risk groups. When considering the infiltration patterns of immune cells, we found higher proportions of follicular helper T (Tfh) cell and M1 macrophage, while lower infiltration of M0 macrophage in low-risk groups (*P* < 0.05). Accordingly, TMB levels of low-risk patients were significantly higher both in OS and DFS model (*P* < 0.01).

**Conclusions:** Our TRGs-based models are reliable predictive tools for OS and DFS. High TMB may confer with an immunogenic microenvironment and predict favorable outcomes in EOCs.

## Introduction

Epithelial ovarian cancer (EOC) is one of the leading causes of cancer death in women, with the 5-year survival for all stages estimated at 45.6%. This rate increases to more than 70% in the minority of patients who are diagnosed at an early stage, but declines to 35% in the vast majority of patients diagnosed at advanced stage 1. Traditional surgery and adjuvant therapies have limited function to improve the prognosis of advanced EOC. Immunotherapy, especially treatment with immune checkpoint inhibitors has been a fast-moving field of clinical cancer research, and promising results of anti-PD-1-antibodies had been confirmed in recurrent EOC 2-4. However, the reported overall response rate of anti-PD-1-antibodies was only 10.7%-25% 4-6. Selecting patient groups that particularly benefit from immunotherapy is desiderated, but also clinically challenging as predictive biomarkers are lacking 7. Further insights are desperately needed to predict the outcome of EOC to improve the survival rate.

Tumor mutational burden (TMB), the number of somatic mutations per DNA megabase (Mb), has emerged as a novel biomarker of response to immunotherapy, with higher TMB inclining to harbor more neoantigens as targets for activated immune cells 8. The positive relationship between TMB and response to CTLA-4 and PD-1 inhibition has been demonstrated in melanoma and non-small cell lung cancer 9, 10. There is growing evidence that ovarian cancers with a higher somatic mutation burden also respond better to cytotoxic chemotherapy 11. Since the TMB scores observed in EOC are lower than that in other carcinomas, it remains unclear that whether patients with a relatively high TMB could benefit more from immunotherapy or have favorable survival outcomes.

In the present study (Fig S1), we investigated the relationship of TMB and prognosis of EOC using two TMB-related prognostic signatures, which were constructed with the somatic mutation and gene expression data from The Cancer Genome Atlas (TCGA). The independent prognostic power of that two models was confirmed by time-dependent receiver operating characteristic (ROC), Kaplan-Meier curve and multivariate Cox regression analysis. Furthermore, the differential abundance of immune cell infiltration, expression levels of immunomodulators and functional enrichment in high- and low-risk groups were also analyzed to understand the underlying mechanism in immunoregulation.

## Materials and methods

### Data collection

We downloaded the somatic mutation data from TCGA database via the GDC data portal (https://portal.gdc.cancer.gov/), and selected the “Masked Somatic Mutation” data and processed it based on the VarScan software. We prepared the Mutation Annotation Format (MAF) of somatic variants and used “maftools” package in R software for the visualization and analysis of mutated spectral data. Besides, transcriptome profiles with HTSeq-FPKM format and clinical information including age, FIGO stage, tumor grade were all obtained from TCGA database (Table S1).

### Calculation of TMB and clinical data analysis

To calculate TMB, the total number of mutations counted is divided by the size of coding region of the targeted territory. We divided the cases into high‐ and low‐TMB group using the median as cut-off value. Then the TMB levels from TCGA cohort were merged with corresponding survival data of each sample via merge function in R. Kaplan-Meier analysis was conducted to compare the difference of OS and DFS between high‐ and low‐TMB group. Correlations between TMB and clinical data were analyzed by Wilcoxon rank-sum test as well.

### Differentially expressed genes and pathway analysis

The “limma” package was used to find the differentially expressed genes (DEGs) between high‐ and low‐TMB group. FDR < 0.05 were taken as the cutoff criterion. Gene Ontology (GO) and Kyoto Encyclopedia of Genes and Genomes (KEGG) analysis of DEGs were implemented using “ggplot2” and “ClusterProfiler” packages.

### Construction and validation of TMB-related prognostic signatures

OS and DFS were chosen as two important outcomes of EOC. Univariate Cox proportional hazards regression (CPHR) analysis was utilized to select the prognostic genes from TRGs with *P* < 0.05. Candidate genes associated to a great extent with OS and DFS were assessed using Least Absolute Shrinkage and Selection Operator (LASSO) method, the coefficients and partial likelihood deviance were calculated with “glmnet” package in R. Multivariate CPHR analysis was further used to construct the prognostic signatures. Samples were classified into high- and low-risk group according to the median risk score, then, the survival difference was compared using the R package “survival”. Time-dependent ROC curves were drawn to evaluate the predictive power of the prognostic signatures in both training and entire cohort by “timeROC” package. Moreover, a conjoint CPHR analysis was applied to define the independent prognostic variables of OS and DFS. Subgroup survival analyses stratified by clinicopathological factors were also conducted.

### Nomograms

Prognostic nomograms that incorporated the TRGs signature and clinical risk factors were constructed by “rms” package to provide a quantitative method for individualized survival prediction. Prognostic accuracy of the nomograms at 1,3,5-years was compared and demonstrated by ROC and calibration curves.

### Gene set enrichment analysis (GSEA)

The collection of annotated gene sets of c2.cp.kegg.v6.0.symbols.gmt in Molecular Signatures Database (MSigDB, http://software.broadinstitute.org/gsea/msigdb/index.jsp) was chosen as the reference gene sets in GSEA software. *P* < 0.05 were chosen as the cutoff criterion.

### Immune cell infiltration and immune checkpoints

CIBERSORT is a newly developed algorithm for characterizing fractions of cell subsets using gene expression data obtained from bulk samples. We estimated the infiltration of 21 immune cell types (infiltration of naive CD4+ T cell was 0 in all samples) in low- and high-risk groups using CIBERSORT, with violin plots showing the distinct compositions. Then, we analyzed the relationship between 21 immune cell types and 17 crucial immune checkpoint modulators (including B7-H3, B7-H4, CD27, CD270, CD40, CD58, CD70, CD86, CTLA4, ICOS, IDO1, LAG3, PD-1, PD-L1, PD-L2, TIGIT, and TIM-3) in EOC sample 12-14. The expression of immunomodulators in high- and low-risk group was also investigated.

### TMB profiles

Wilcoxon rank-sum test was conducted to assess the TMB level of EOC samples in high- and low-risk groups. Mutation type, the top 10 mutated genes, associations across mutate genes in these groups were analyzed using “maftools” package.

### Statistical analysis

Univariate, multivariate CPHR and LASSO analyses were executed to construct the TMB-related prognostic signatures, whose predictive capacities were evaluated using time-dependent ROC analysis. Conjoint CPHR analysis was applied to define the independent prognostic variables of OS and DFS, and the predictive accuracy of each variable was tested via time-dependent ROC analysis. Survival curves were estimated by Kaplan-Meier method and compared by the log-rank test. *P* < 0.05 was determined statistically significant. All analyses were performed using R (version 3.3.1) and Bioconductor.

## Results

### Genome‐wide mutation profiling in EOC

**Figure 1:**
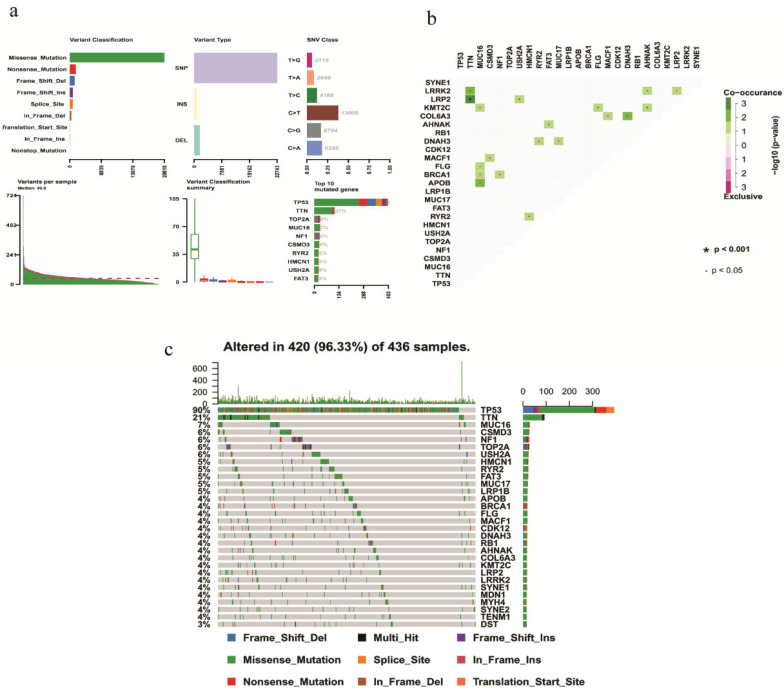
** Summary of mutation profiling in EOC samples. a** Distribution of variants based on variant classification, type, and SNV class. Bottom part (from left to right) indicates mutation load for each sample, and the top 10 mutated genes in EOC. **b** The coincident and exclusive associations across mutated genes displayed as a triangular matrix. **c** Landscape of mutation profiles was shown in the waterfall plot, in which the type of mutation is shown in the comment bar (bottom) and genes are ordered by their mutation frequency.

On the whole, various mutation categories were summarized in different groups, where missense mutation was the most common type. Single‐nucleotide polymorphism occurred more frequently than deletion or insertion, and C >T transition accounted for the largest part in the SNV classification. Horizontal histogram revealed the top 10 mutated genes in EOC, including *TP53* (90%), *TTN* (21%), *MUC16* (7%), *TOP2A* (6%), *NF1* (6%), *CSMD3* (6%), *USH2A* (6%), *RYR2* (5%), *HMCN1* (5%) and *FAT3* (5%) (Fig. 1a). The coincident and exclusive associations across mutated genes were also investigated. For example, mutant *MUC16* coexisted with mutant *KMT2C, FLG, BRCA1*, and *APOB* significantly (*P* < 0.05) (Fig. 1b). The waterfall plot showed details of mutation in 420 samples, where various colors with annotations at the bottom represented different mutation types (Fig. 1c).

### TMB correlated with survival outcomes and clinical stages

**Figure 2:**
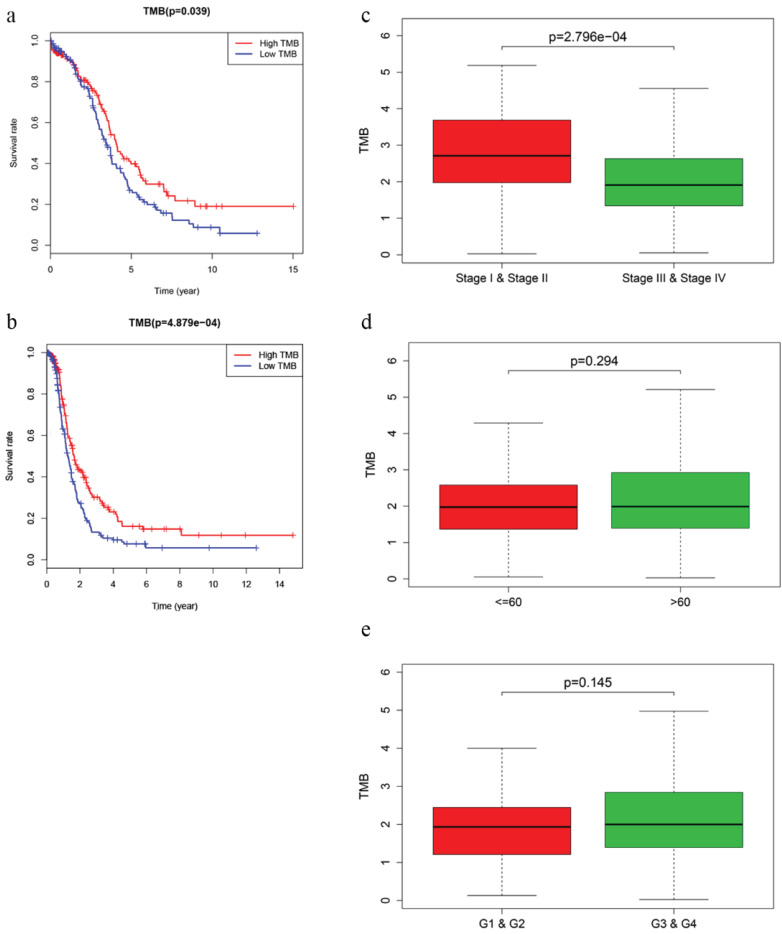
Prognosis of TMB and associations with clinical characteristics. **a, b** Higher TMB level revealed better OS (P = 0.039) and DFS (*P <* 0.001). c-e Higher TMB level correlated with earlier clinical stages with p = 2.796e-04, while no significant difference was observed in patient age or tumor grades.

After calculating the TMB value, we divided EOC patients into high‐ and low‐TMB groups using the median TMB as cut-off value. Kaplan-Meier analysis with log‐rank test indicated that patients in high-TMB group revealed better OS (*P* = 0.039) and DFS (*P* = 4.879e-04) than those in low-TMB group (Fig. 2a, b). Higher TMB level also correlated with earlier clinical stages, while no significant difference was observed in associations with patient age or tumor grades (Fig. 2c-e).

### DEGs identification and functional analysis

A total of 139 genes were identified as differentially expressed TRGs with FDR < 0.05 (Fig S2a). Next, we conducted the GO enrichment analysis, which is composed of three parts: biological process (BP), cellular component (CC), and molecular function (MF). In BP group, regulation of chromosome segregation, ribosomal subunit export from nucleus and maintenance of cell polarity were enriched. In CC group, these DEGs were mainly involved in mitochondrial inner membrane, chromosome region. In MF group, the significantly enriched terms were cytoskeletal adaptor activity, DNA replication origin binding and exo-alpha-sialidase activity (Fig. S2b-d). Besides, KEGG signaling pathway analysis suggested that cell cycle was the most significant pathway (Fig. S2e).

### Development and assessment of the TMB-related prognostic signatures

We integrated the transcriptome and clinical data so as to screen out 351 OS-related and 319 DFS-related prognostic EOC samples. For OS, training cohort composed of 176 randomly-selected samples were used to construct the prognostic signature, while the entire cohort was used to validate the predictive power. Similarly, the DFS signature was constructed based on 160 samples. After step-by-step gene selection through univariate CPHR, LASSO regression and multivariate CPHR analysis, finally, seven genes (*B3GALT1, LIN7B, ANGPT2, D2HGDH, TAF13, PFDN4* and *DNAJC19*) were confirmed as the most OS-related genes, and the four genes (*LPIN3, PXYLP1, IGSF23* and *B3GALT1*) were identified as the most DFS-related genes for EOC patients. Risk score for OS = 0.17849 * *B3GALT1* + (-0.15850) * *LIN7B* + (-0.20095) * *ANGPT2* + 0.06441 * *D2HGDH* + (-0.03555) * *TAF13* + 0.03600 * *PFDN4* + (-0.05400) * *DNAJC19*. Risk score for DFS = 0.06416 * *LPIN3* + (-0.20524) * PXYLP1 + (-0.09458) * *IGSF23* + 0.18717 * *B3GALT1* (Table 1,2,3,4).

**Figure 3:**
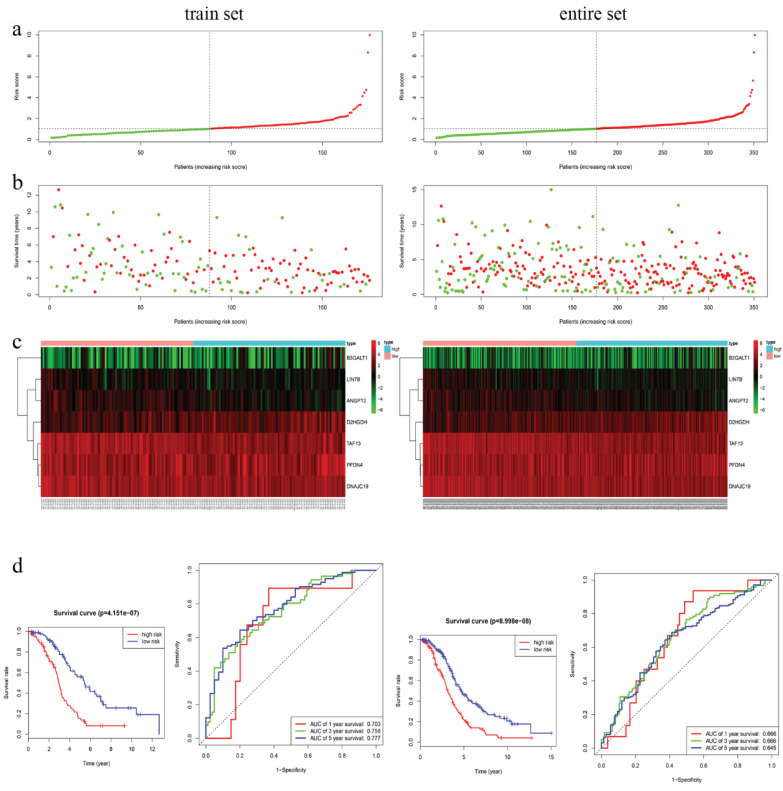
Characteristics of the 7-gene prognostic signature in the training and entire cohort. **a** The risk score of each EOC patient; **b** OS and survival status of the patients; **c** Heat maps of gene expression profiles; **d** Left panel: Kapan-Meier curves suggested thar EOC patients in low-risk group had much better OS than those in the high-risk group (*P* < 0.001). **d** Right panel: Time-dependent ROC curves at 1 year, 3 years and 5 years based on the7-gene signature.

In training cohort, we calculated the seven-gene based risk score for each patient, and then separated the samples into high- (n=88) and low-risk group (n=88) using the median value as cutoff point. Patients in entire cohort were also separated into high- (n=174) and low-risk group (n=177) according to the same cutoff value. The distribution of risk scores, OS, OS status, and expression of seven genes in the cohorts were showed in Fig. 3a-c. Kapan-Meier survival curves of the training and entire cohort suggested the same result: EOC patients in the low-risk group had much better OS than those in the high-risk group (*P* < 0.001). To assess the predictive performance of the seven-gene based signature, we conducted a time-dependent ROC curve analysis by comparing the respective AUC value. The AUC values of the seven-gene prognostic signature at 1 year, 3 years, and 5 years were 0.703, 0.758 and 0.777 in the training cohort, while 0.666, 0.666, 0.645 in the entire cohort, accordingly (Fig. 3d)**.**

As for DFS model, patients in training cohort were allocated into high- (n=80) and low-risk group (n=80) according to the median value calculated based on the four-gene prognostic signature, with the entire cohort grouping into high- (n=144) and low-risk (n=175). Distribution of risk score, DFS, DFS status, and prognostic-gene expression were showed in Fig S3a-c. Kapan-Meier survival curves showed patients with low risk scores had longer DFS in both cohorts (*P* < 0.01). AUC values of the four-gene prognostic signature at 1 year, 3 years, and 5 years were 0.617, 0.756, and 0.731 in the training cohort, while 0.608, 0.670, 0.778 in the entire cohort, respectively (Fig. S3d)**.**

Furthermore, we performed subgroup analysis of the two signatures in age (≤ 60, > 60), tumor grade (G1 & G2, G3 & G4) and clinical stage (stage I & II, stage III & IV), which suggested that patients with high‐risk scores had shorter OS and DFS in subgroups of age ≤ 60, age > 60, G3 & G4 and stage III & IV (*P* <0.05) (Fig S4). Heatmaps showed expression profiles of the prognosis-related genes in high- and low-risk groups. In OS model, the differential expression was significantly associated with age, while no significant association was found in DFS model (Fig S5).

To assess whether the signature was an independent predictor of EOC, we analyzed the relationships between OS and clinical factors by CPHR model. Multivariate analysis showed that only the signature-based risk score was significantly associated with OS (*P* < 0.05) (Table S2). Risk score was also proved to be an independent prognostic predictor of DFS (Table S3).

**Figure 4:**
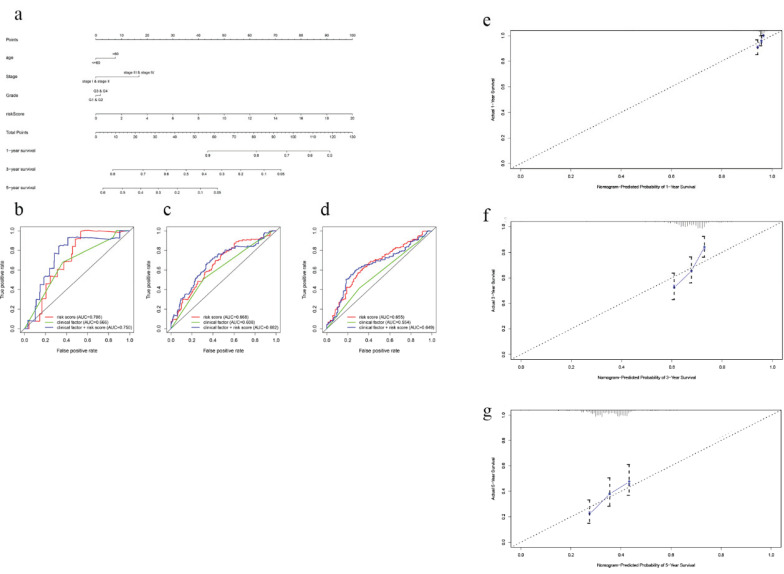
Nomogram to predict OS in EOC patients. **a** Nomogram based on the 7-gene signature and clinical factors for 1-, 3- and 5-year OS prediction. **b** Time-dependent ROCs for the nomogram, clinic factors including age, tumor grade and clinical stage. **c** Calibration plots of the gene-based prognostic model.

In order to establish a clinically applicable method for predicting the survival probability of EOC patients, nomograms including the signature-based risk score and clinical factors were developed (Fig. 4a, Fig S6a). By drawing time-ROC curves, we found that the gene-based signatures had better predictive ability for 1 year-, 3 years-, 5 years-OS and DFS as compared with other clinical factors. Intriguingly, when combining risk score with clinical factors for analysis, the AUC values of 1 year-, 3 years-OS and DFS increased further (Fig. 4b-d, Fig S6b-d), which suggested the nomograms had superior predictive capacity for short-term prognosis. Calibration plots also verified good calibration ability of the signature-based nomograms (Fig. 4e-g, Fig S6e-g).

### Identification of the prognostic signatures related biological pathways

**Figure 5:**
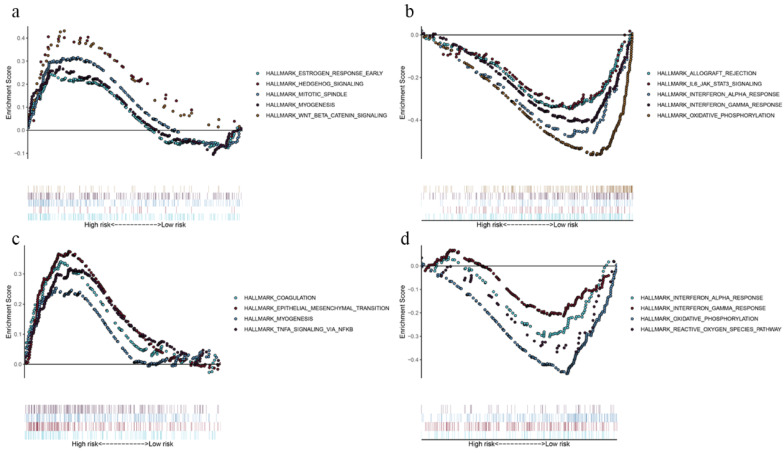
Functional enrichment of the prognostic genes of OS and DFS with GSEA. Pathway enriched in high-risk group of OS (**a**), low-risk group of OS (**b**), high-risk group of DFS (**c**), and low-risk group of DFS (**d**). *P* < 0.05.

By comparing the biological processes enriched in high- and low-risk groups, we defined the underlying biological function of the prognostic genes. In OS model, gene sets for “estrogen response”, “hedgehog signaling”, “mitotic spindle”, “myogenesis” and “Wnt/β-catenin signaling” were enriched in high-risk group (Fig. 5a), while “allograft rejection”, “IL-6-JAK-STAT3 signaling”, “interferon response” and “oxidative phosphorylation” pathways were enriched in low-risk group (Fig. 5b). As for DFS model, gene sets for “coagulation”, “epithelial mesenchymal translation”, “myogenesis” and “TNFA signaling via NF-kB” were enriched in high-risk group (Fig. 5c), while “interferon response”, “oxidative phosphorylation” and “reactive oxygen species pathway” were enriched in low-risk group (Fig. 5d). The GSEA results demonstrated that immune‐related pathways were enriched in low‐risk groups. Therefore, we suggested that the TMB‐related risk signatures may demonstrate an intensive immune phenotype.

### Differential immune cells infiltration in high- and low-risk groups

**Figure 6:**
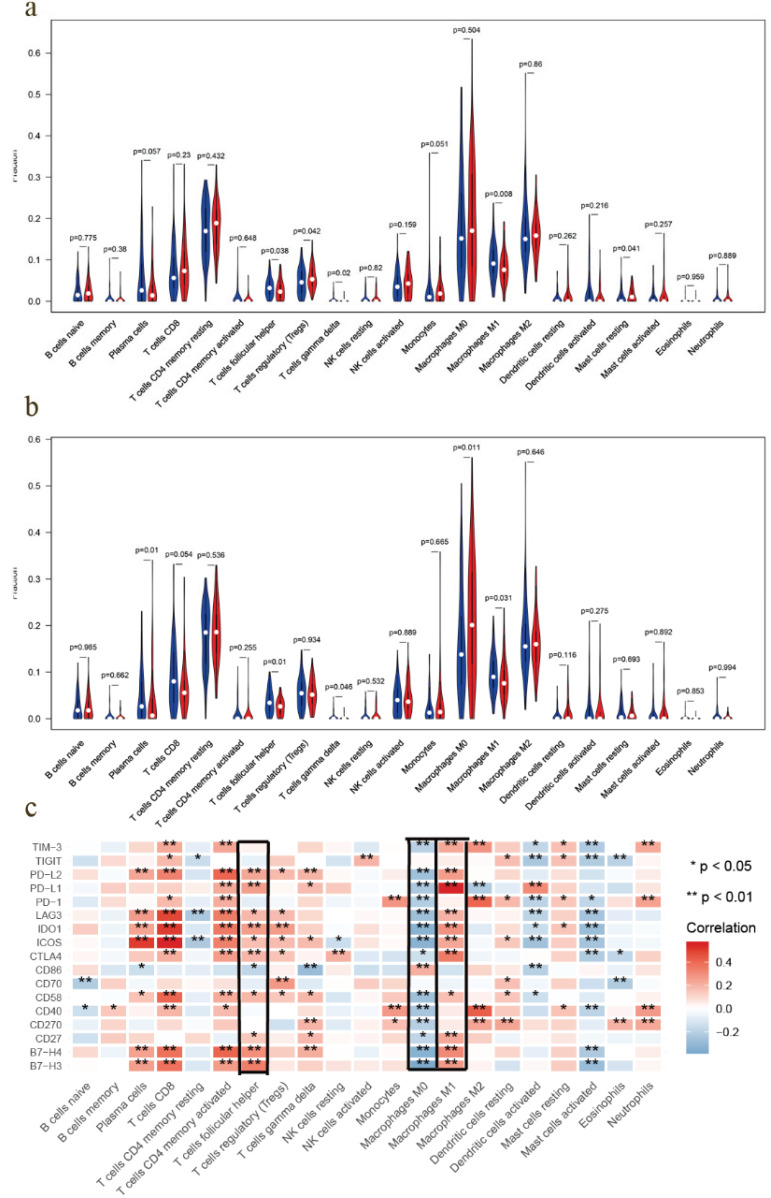
TIC distribution and the correlation between immunomodulators. **a** EOC samples in low-risk group (based on the OS-signature) had higher proportions of follicular helper T (Tfh) cell, γδ T cell and M1 macrophage, but lower proportions of T-reg and resting mast cell (*P* < 0.05). **b** Samples in low-risk group (based on the DFS-signature) had higher proportions of plasma cell, Tfh cell, γδ T cell and M1 macrophage, but lower proportion of M0 macrophage (*P* < 0.05). **c** Correlations between immunomodulators and TICs, which showed Tfh cell and M1 macrophage correlated positively with most modulators, while M0 macrophage did negatively.

**Figure 7:**
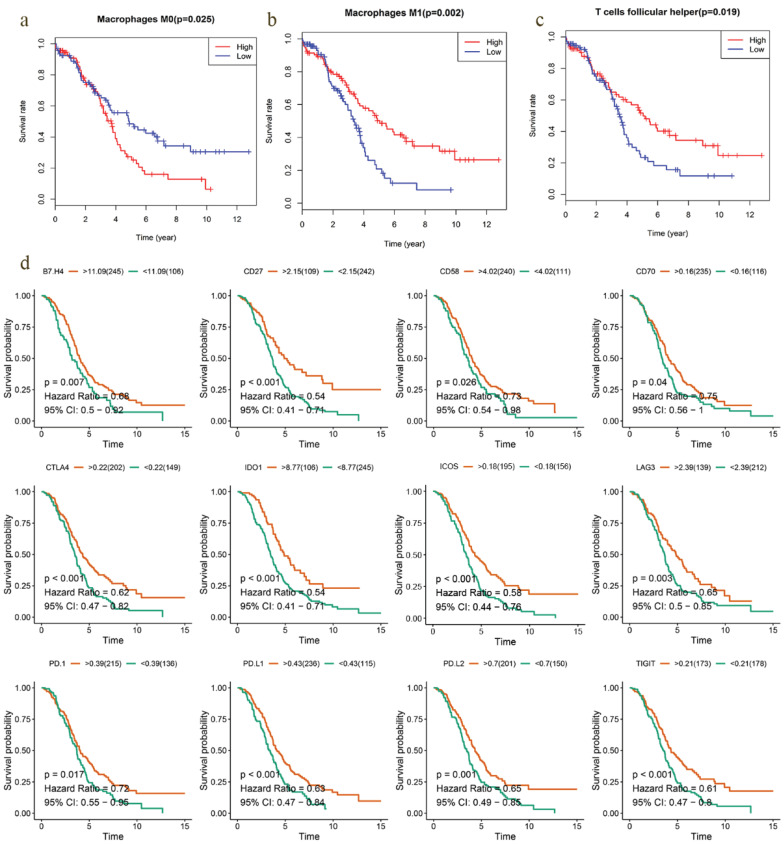
Survival analysis of TICs and immunomodulators. **a-c** Kaplan-Meier curves showed that Tfh cell and M1 macrophage correlated with favorable survival outcome, but M0 macrophage correlated with poor survival in EOC. **d** High expression of B7-H4, CD27, CD58, CD70, CTLA4, ICOS, IDO1, LAG3, PD1, PD-L1, PD-L2 and TIGIT was shown to be associated with favorable survival outcome (*P* < 0.05).

Most solid tumors are infiltrated by myeloid- and lymphoid lineage-derived immune cells that are differentially distributed within the tumor microenvironment (TME) with a crucial role in the establishment of antitumoral responses or tumor progression 15. Thus, composition profiles of immune cells in high- and low-risk samples were identified and shown by the violin plots. Compared with the high-risk group (divided by OS-signature), samples in low-risk group generally contained higher proportions of follicular helper T (Tfh) cell, γδ T cell and M1 macrophage, but lower proportions of regulatory T cell (T-reg) and resting mast cell (*P* < 0.05) (Fig. 6a). As to DFS-signature, infiltrating levels of plasma cell, Tfh cell, γδ T cell and M1 macrophage were higher in low‐risk group, while that of M0 macrophage was lower (*P* < 0.05) (Fig. 6b). Furthermore, Tfh cell and M1 macrophage correlated with the survival rate positively, while M0 macrophage did negatively (Fig. 7a-c).

### Prognostic value of immune checkpoint modulators and the correlation with immune cells

Previous studies have discovered that immune checkpoint modulators mediate the function of tumor infiltrating cells 16. Therefore, we evaluated the correlation of immunomodulators and immune cells in EOC, which suggested that Tfh cell and M1 macrophage correlated positively with most modulators, and M0 macrophage did negatively (Fig. 6c). We also assessed the predictive performance of immunomodulators in EOC. Kaplan Meier curves demonstrated that high expression of B7-H4, CD27, CD58, CD70, CTLA4, ICOS, IDO1, LAG3, PD1, PD-L1, PD-L2 and TIGIT was associated with favorable survival significantly (Fig. 7d). Meanwhile, the differential expression profiles of immunomodulators indicated an immunogenic TME in low-risk EOC samples (Fig S7).

### Mutation profiles of high- and low-risk group

**Figure 8:**
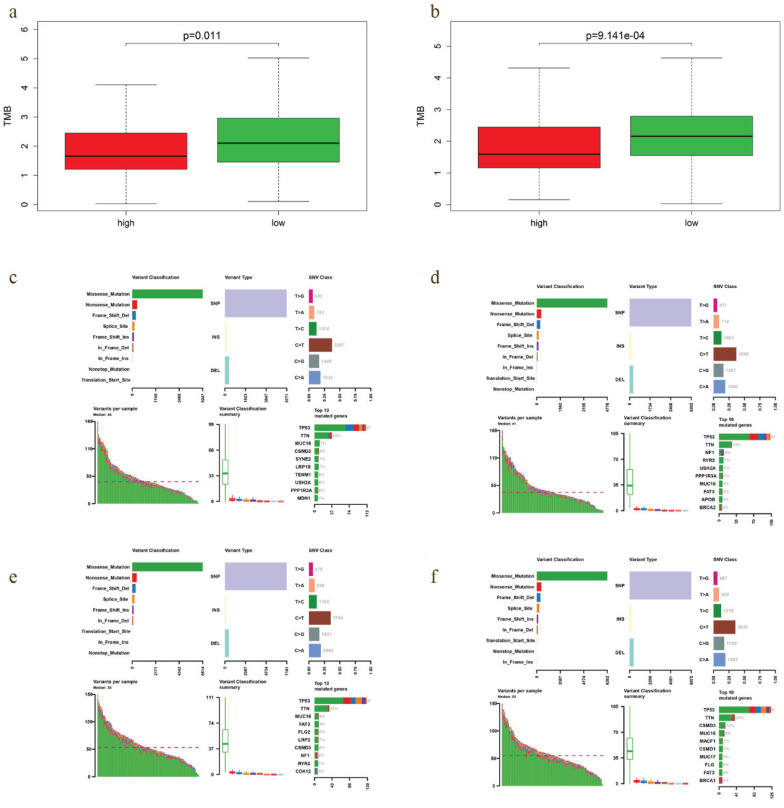
TMB level and mutation profiling in EOCs with different prediction of survival. **a-b** TMB level of low-risk group was significantly higher in both OS (*P* = 0.011) and DFS model (P = 9.141e-04). **c, e** Mutation profiling of EOCs with high- and low-risk in OS model, respectively. **d, f** Mutation profiling of cases with high- and low-risk in DFS model, respectively.

A positive relationship between TMB and immunotherapy responsiveness had been reported in non-small cell lung cancer, melanoma, bladder and breast cancer patients 17-19. Our study demonstrated that TMB correlated closely with the survival outcomes of EOC. Therefore, we compared the TMB profiles in low- and high-risk group to further verify the relationship between TMB and prognosis. Interestingly, TMB level of low-risk group was significantly higher in both OS (*P* = 0.011) and DFS model (*P* = 9.141e-04) (Fig. 8a, b), with the top 4 mutated genes including *TP53, TTN, CSMD3* and *MUC16* (Fig. 8e, f).

## Discussion

A molecular marker-based approach to accurately predict outcomes of EOC patients is urgently needed in the era of precision medicine. TMB has been confirmed closely linked to the prognosis of cancer patients. Federico et al. 20 demonstrated that low TMB was one of the negative prognostic factors in patients with metastatic colorectal cancer treated in first line. Zhang et al. 8 reported that molecular subtyping based on TMB is a potential prognostic marker for lung adenocarcinoma. Nicolai et al. 21 found that TMB coupled with *BRCA1* or *BRCA2* mutations in ovarian cancer was a genomic marker of prognosis and predictor of treatment response. Similarly, based on the results of survival analysis, our study showed that patients in high-TMB group had better survival outcomes than those in low-TMB group, and DEGs between the two groups were identified for deep investigation.

Multiple biostatistics methods were applied to select the prognostic genes from TRGs and construct signatures to improve outcomes prediction. Seven OS-related genes (*B3GALT1, LIN7B, ANGPT2, D2HGDH, TAF13, PFDN4, DNAJC19*) and 4 DFS-related genes (*LPIN3, PXYLP1, IGSF23, B3GALT1*) were detected as independent prognosis predictors in EOC. *B3GALT1*, the common gene of the prognostic signatures, is a member of the beta-1,3-galactosyltransferase (β3GalT) gene family, which encodes type II membrane-bound glycoproteins and plays an essential role in the O-glycosylation process. Since glycoproteins rich in O-glycosylation domains are expressed on the surface of cancer cells, β3GalT family may be closely related to tumors 22. Chachadi et al. 23 confirmed that B3GALT1 participated in the synthesis of Sialyl Lewis antigens, which were involved in metastasis of prostatic cancer cells. *ANGPT2*, also named angiopoietin-2 (Ang-2), was shown to be correlated with poor prognosis in breast, hepatocellular, colorectal and prostate cancer 24-27. Prefoldin 4 (PFDN4) is a transcriptional factor that regulates the cell cycle, further, Miyoshi et al. 28 revealed that patients with high expression of *PFDN4* showed a better prognosis for overall survival in colorectal cancer. Yet, for other genes, there are few studies about their roles in tumor prognosis.

Our study showed that patients in low-risk group had better OS and DFS than those with high risk scores in both training and entire cohort. Hence, these 2 TRG signatures were of prognostic significance. AUC values of the signatures at 1 year-, 3 years-, and 5 years in training and entire cohorts ranged from 0.608 to 0.778, thus, large-sample researches are needed for further verification and modification before clinical application.

Functional enrichment analysis demonstrated that immune‐related pathways were enriched in low‐risk groups. Among the differently infiltrated immune cells, Tfh and M1 macrophage infiltration was found to be correlated with favorable survival outcomes, while M0 macrophage correlated with poor survival in EOC cases. Similar results were demonstrated in other tumors, Tfh-related cells had been positively associated with long-term survival of humans with breast and colorectal cancer 29, 30. In addition, Shane Crotty 31 suggested the hypotheses for the value of Tfh cells in immune responses against tumors: (1) Tfh may help develop or support ectopic lymphoid structures, which are a site of recruitment for CD8+ T cells, NK cells, and macrophages that engage in anti-tumor immunity. Alternatively, (2) Tfh cells may support anti-tumor Ab responses by B cells. Macrophages are one of the most abundant immune cells in TME of solid tumors and their presence correlates with reduced survival in most cancers 32. High population of macrophages is associated with poor prognosis in ovarian cancer and could impact the efficacy of immune therapies 33, 34. Macrophages display a high plasticity, and adapt their phenotype in response to different environmental stimuli. The density of M1 macrophage in tumor islets was found to be positively associated with survival time in non-small cell lung cancer patients 35. Interferon-γ could induce the differentiation of M1 macrophages, and the M1 macrophages produce high levels of interleukin (IL)-12, IL-23, TNF-a, IL-1, IL-6, reactive oxygen and nitrogen intermediates, as well as other effector molecules 36-38. Therefore, we hypothesized that the enriched “interferon response”, “IL-6 signaling” and “reactive oxygen species pathway” in low-risk groups may related to the infiltration of M1 macrophages.

Usually, the complexity of tumor-infiltrating immune cells (TICs) broadly affects the host immune status and plays an important role in the response to immunotherapy. Previous studies have discovered that immune checkpoint modulators could mediate the function of TICs 39. In our study, we found that Tfh cell and M1 macrophage correlated positively with most modulators, while M0 macrophage did negatively. And high expression of most modulators was associated with favorable outcomes in EOC. Hence, we speculated that there was an immunogenic TME in low-risk samples, which may be one of the reasons for better survival. The speculation was further confirmed by the high expression of modulators in low-risk groups.

Higher TMB level correlated with improved OS and DFS, meanwhile, TMB levels of low-risk patients were significantly higher in both OS and DFS model. Therefore, we regarded TMB as a good prognostic marker for EOC. Since TMB is positively correlated with the response to immunotherapy, and there could be an immunogenic TME in low-risk samples, we deduced that immunoregulation may be involved in the prognosis prediction of TMB in EOC.

Then, we further explored the TMB profiles, and found *TP53, TTN, FAT3, CSMD3, MUC16* and *FLG* were among the top 10 mutated genes of low-risk group in both OS and DFS model. The most predominant genetic alternation in EOC is the mutation of *TP53*, which had been considered as diagnostic and prognostic biomarker as well as therapeutic target for EOC 40. Wieser et al. 41 found that TP53 mutation might serve as a potential predictive factor of anti-PD1/PD-L1 immunotherapy in ovarian cancer. *MUC16* encodes a repeating peptide epitope of mucin that promotes cancer cell proliferation and inhibits anti-cancer immune responses 42. Filaggrin (*FLG*), was found to identify a distinct patient population with low immune cell infiltration in melanoma and ovarian cancer, and may protect cancer cells from immune cell infiltration and immune-mediated destruction 43. Mutations of *FAT3, CSMD3* and *TTN* have been reported in EOC 44, 45, but their influence on immunoregulation was still unknown.

However, there are also some limitations to our study: (i) basic experiment is needed to validate the association between genes signatures and immune infiltrates; (ii) lack of external validation; (iii) further studies with lager sample size are required in the future.

## Conclusion

We constructed and validated 2 TMB-related prognostic signatures, which may act as promising prognostic molecular biomarkers and have immunotherapeutic implications for EOC patient management.

## Supplementary Material

Supplementary figures and tables.

## Figures and Tables

**Table 1:** Univariate cox analysis of TMB-related signature with OS

Gene	HR	95%CI	*p* value
ACSS3	1.422	1.173-1.723	0.000
ANGPT2	0.782	0.654-0.935	0.007
HAPLN1	0.622	0.427-0.904	0.013
D2HGDH	1.065	1.012-1.120	0.016
TAF13	0.962	0.931-0.993	0.018
RARG	1.024	1.003-1.045	0.024
LPCAT4	1.046	1.006-1.089	0.025
DNAJC19	0.964	0.932-0.996	0.029
PFDN4	1.018	1.002-1.034	0.030
B3GALT1	1.160	1.009-1.333	0.037
PGM3	0.907	0.825-0.997	0.043
SELENOT	0.986	0.973-1.000	0.044
GCH1	0.886	0.786-0.998	0.046
LIN7B	0.817	0.669-0.997	0.047
LTA4H	1.029	1.000-1.059	0.050

**Table 2:** Multivariate Cox analysis of TMB‐related signature with OS

Gene	description	coef	HR	*p* value
B3GALT1	Beta-1,3-galactosyltransferase 1	0.17849	1.20 (1.04-1.37)	0.01026
LIN7B	Lin-7 homolog B	-0.15850	0.85 (0.69-1.05)	0.13512
ANGPT2	Angiopoietin 2	-0.20095	0.82(0.69-0.98)	0.02504
D2HGDH	D-2-hydroxyglutarate dehydrogenase	0.06441	1.07(1.01-1.13)	0.02468
TAF13	TATA-box binding protein associated factor 13	-0.03555	0.97(0.93-1.00)	0.05265
PFDN4	Prefoldin subunit 4	0.03600	1.04(1.02-1.05)	0.00001
DNAJC19	DnaJ heat shock protein family (Hsp40) member C19	-0.05400	0.95(0.91-0.98)	0.00482

**Table 3:** Univariate cox analysis of TMB-related signature with DFS

Gene	HR	95%CI	*p* value
PXYLP1	0.832	0.724-0.956	0.009
LPIN3	1.065	1.013-1.120	0.014
GCH1	0.862	0.763-0.973	0.016
B3GALT1	1.182	1.029-1.356	0.018
CDC6	0.884	0.796-0.982	0.021
C3orf38	0.921	0.855-0.993	0.032
NOCT	0.846	0.724-0.988	0.035
RASSF7	1.008	1.001-1.015	0.036
IGSF23	0.913	0.837-0.996	0.041
TRIP6	1.010	1.000-1.020	0.045

**Table 4:** Multivariate Cox analysis of TMB‐related signature with DFS

Gene	description	coef	HR	*P* value
IGSF23	Immunoglobulin superfamily member 23	-0.09458	0.91(0.83-0.99)	0.03392
LPIN3	Lipin 3	0.06416	1.07(1.01-1.12)	0.01644
PXYLP1	2-phosphoxylose phosphatase 1	-0.20524	0.81(0.70-0.94)	0.00612
B3GALT1	Beta-1,3-galactosyltransferase 1	0.18717	1.21(1.04-1.39)	0.01107
