# Supplementary material for: Multi‐omics analysis of tumor mutational burden combined with prognostic assessment in epithelial ovarian cancer based on TCGA database
Source: Int J Med Sci. 2020 Oct 23;17(18):3200–13. doi: 10.7150/ijms.50491 (PMC7646107; doi:10.7150/ijms.50491)
Supplement: Supplementary file 1 — Supplementary figures and tables. [file ijmsv17p3200s1.pdf]

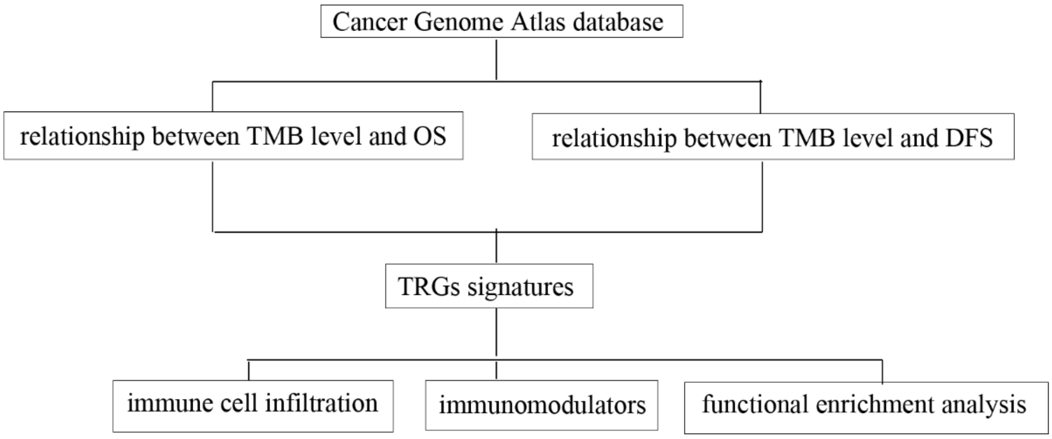

Fig S1 Flow chart of data processing, analysis in this study.

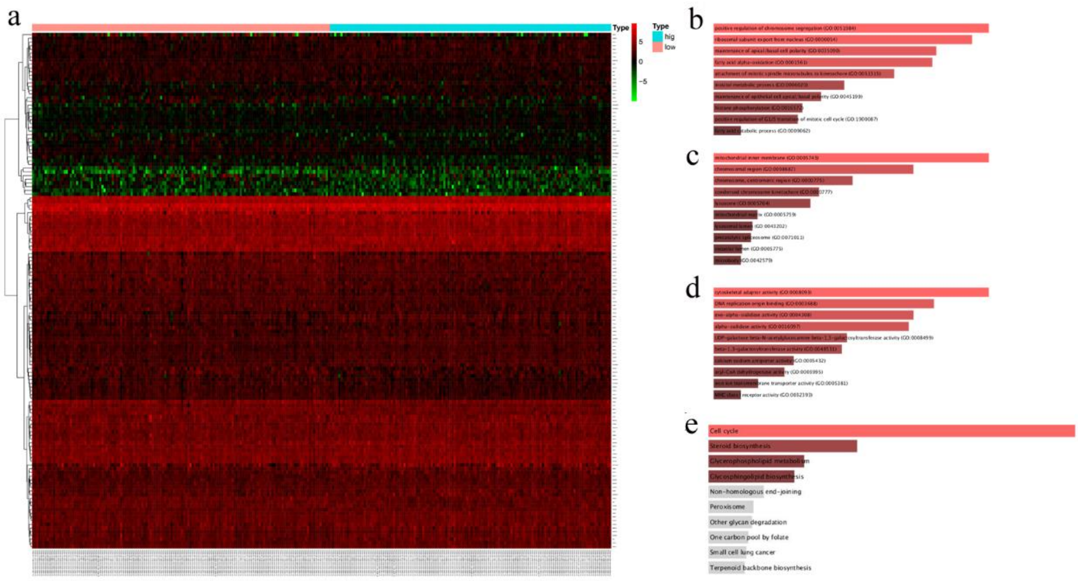

Fig S2 Analysis of differential gene expression and pathway enrichment in high- and low-TMB group. a The heatmap exhibited DEGs in high- and low-TMB group. b-d GO enriched results revealed that the DEGs might be related to chromosome segregation, chromosome segregation, cytoskeletal adaptor activity or other functional pathways. e KEGG analysis suggested that cell cycle was the most significant pathway.

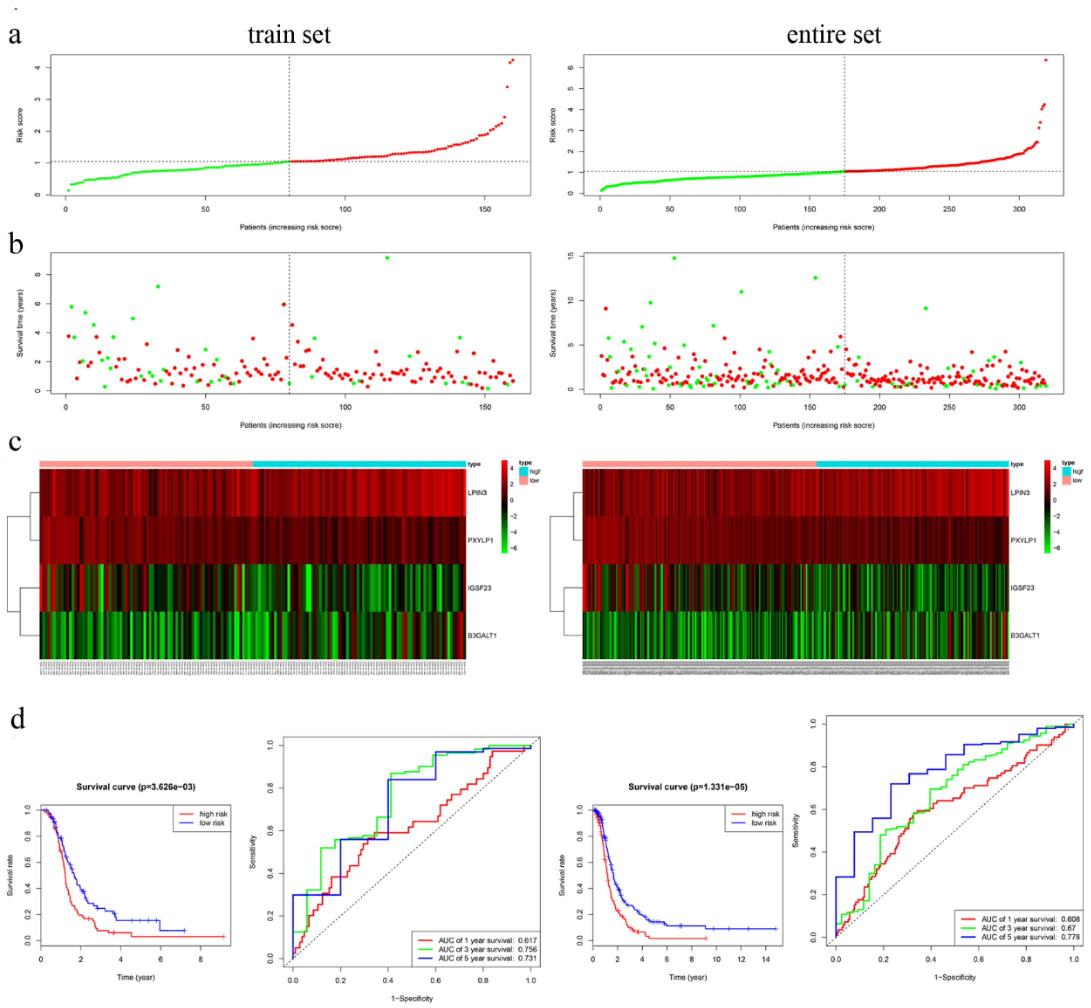

Fig S3 Characteristics of the 4-gene prognostic signature in the training and entire cohort. **a** The risk score of each EOC patient; **b** DFS and survival status of the patients; **c** Heat maps of gene expression profiles; **d** Left panel: Kapan-Meier curves suggested thar EOC patients in low-risk group had much better DFS than those in the high-risk group (P < 0.01). **d** Right panel: Time-dependent ROC curves at 1 year, 3 years and 5 years based on the 4-gene signature.

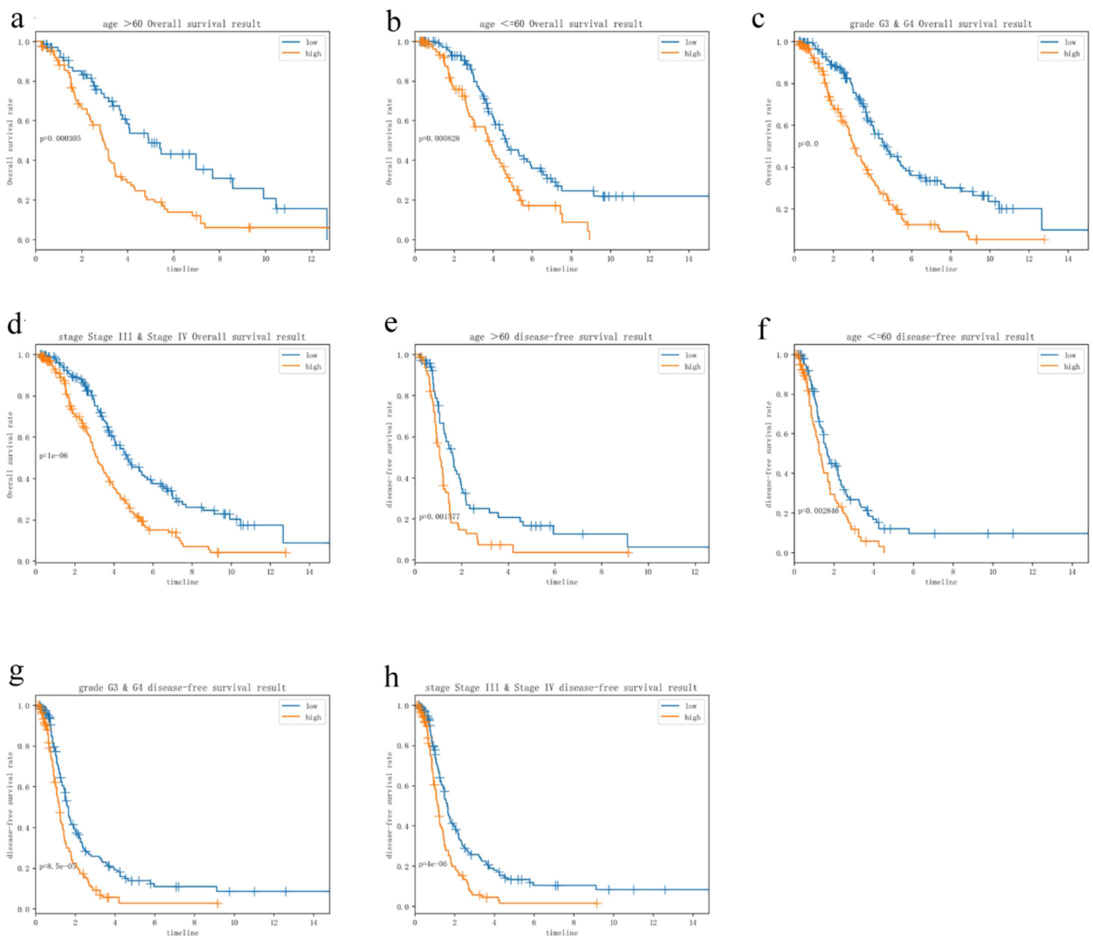

Fig S4 Stratified analysis of the OS- and DFS-signature for EOC patients, which suggested patients with high-risk scores had shorter OS and DFS in subgroups of age ≤ 60, age > 60, G3 & G4 and stage III & IV ($p<0.05$).

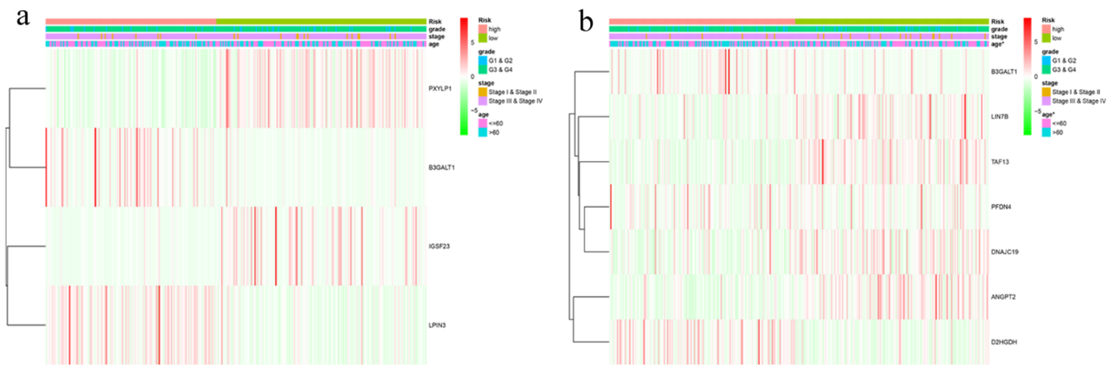

Fig S5 Expression profiles of the prognosis-related genes of OS and DFS in high- and low-risk groups. **a** No significant association was found in DFS model; **b** Differential expression profiles were significantly associated with age in OS model.

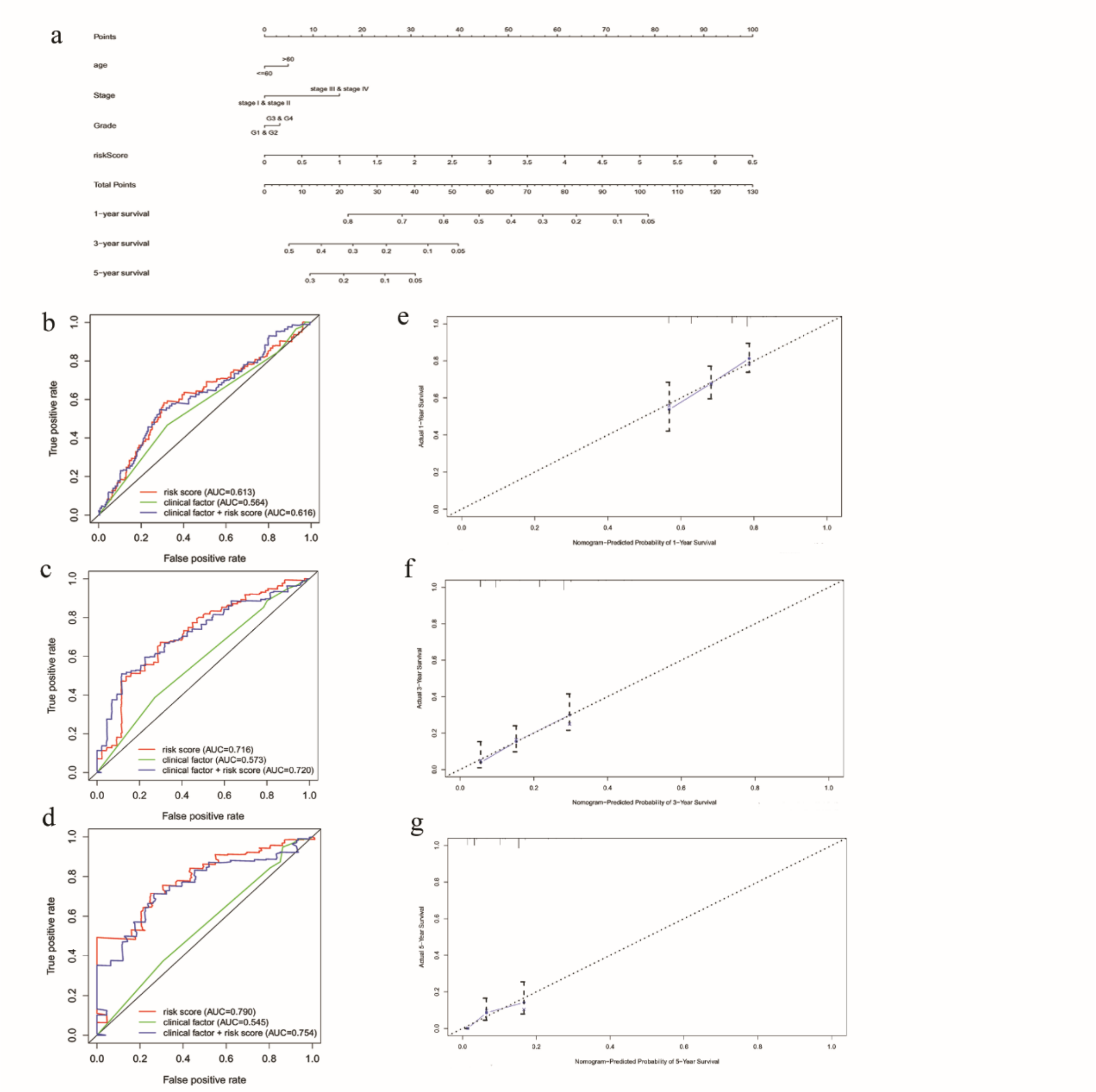

Fig S6 Nomogram to predict DFS in EOC patients. **a** Nomogram based on the 4-gene signature and clinical factors for 1-, 3- and 5-year DFS prediction. **b** Time-dependent ROCs for the nomogram. **c** Calibration plots of the gene-based prognostic model.

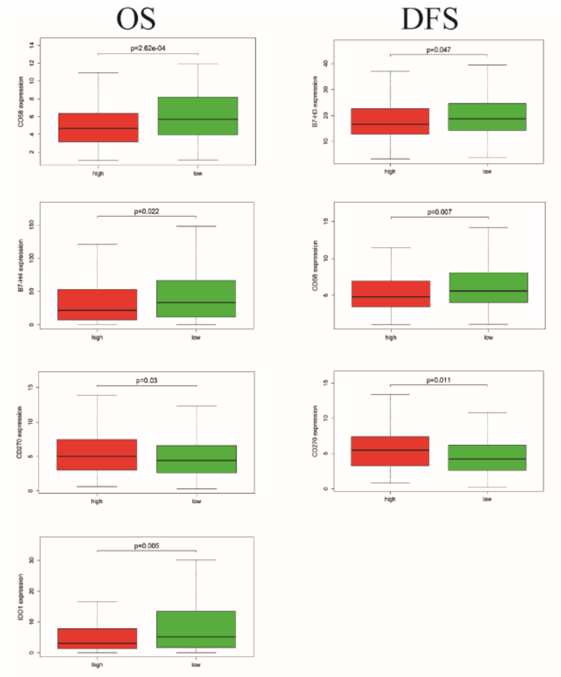

Fig S7 Differential expression profiles of immunomodulators indicated an immunogenic TME in low-risk EOCs. In OS model, the expression of CD 58, B7-H4, IDO1 were significantly higher, but that of CD 270 was lower in low-risk group. In DFS model, the expression of CD 58, B7-H3 were significantly higher, and that of CD 270 was lower in low-risk group.

Table S1 Clinical characteristics of EOC patients

Clinical parameters	Variable	Total (584)	Percentages (%)
Age	≤60	330	56.5
	>60	254	43.49
stage	Stage I & Stage II	50	8.56
	Stage III & Stage IV	534	91.44
grade	G1 & G2	85	14.55
	G3 & G4	499	85.45

Table S2: Univariate and multivariate Cox regression analyses of OS in TCGA training and entire cohort

Variables	Univariate analysis			Multivariate analysis		
	HR	95%CI	*p*	HR	95%CI	*p*
**TCGA training cohort**						
Stage (Stage I & Stage II vs Stage III & Stage IV)	2.41	0.59-9.83	0.22	1.95	0.45-8.38	0.37
Grade (G1 & G2 vs G3)	1.63	0.84-3.15	0.15	1.40	0.71-2.79	0.33
Age (≤60 vs ＞60)	1.32	0.87-2.02	0.20	1.22	0.79-1.88	0.37
Risk Score (high / low)	1.19	1.11-1.28	**0.00**	1.18	1.10-1.27	**0.00**
**TCGA entire cohort**						
Stage (Stage I & Stage II vs Stage III & Stage IV)	1.71	0.70-4.18	0.24	1.67	0.68-4.12	0.27
Grade (G1 & G2 vs G3)	1.11	0.72-1.72	0.64	1.06	0.68-1.65	0.80
Age (≤60 vs ＞60)	1.31	0.98-1.74	0.07	1.26	0.94-1.68	0.12
Risk Score (high / low)	2.05	1.67-2.52	**0.00**	1.16	1.09-1.24	**0.00**

Bold values indicate $P<0.05$.

Table S3: Univariate and multivariate Cox regression analyses of DFS in TCGA training and entire cohort.

Variables	Univariate analysis			Multivariate analysis		
	HR	95%CI	*p*	HR	95%CI	*p*
**TCGA training group**						
Stage (Stage I & Stage II vs Stage III & Stage IV)	5.11	1.26-20.69	**0.02**	4.96	1.21-20.41	**0.03**
Grade (G1 & G2 vs G3)	1.57	0.91-2.72	0.11	1.18	0.68-2.06	0.56
Age (≤60 vs ＞60)	1.02	0.70-1.48	0.92	1.01	0.68-1.48	0.98
Risk Score (high / low)	2.01	1.54-2.62	**0.00**	1.98	1.50-2.60	**0.00**
**TCGA entire group**						
Stage (Stage I & Stage II vs Stage III & Stage IV)	1.61	0.85-3.04	0.14	1.65	0.87-3.13	0.13
Grade (G1 & G2 vs G3)	1.15	0.76-1.73	0.51	1.11	0.73-1.68	0.63
Age (≤60 vs ＞60)	1.24	0.95-1.61	0.12	1.17	0.90-1.53	0.25
Risk Score (high / low)	1.67	1.37-2.04	**0.00**	1.65	1.34-2.02	**0.00**

Bold values indicate $P<0.05$.
